# Internal quality assurance in diagnostic microbiology: A simple approach for insightful data

**DOI:** 10.1371/journal.pone.0187263

**Published:** 2017-11-14

**Authors:** Valentin Scherz, Christian Durussel, Gilbert Greub

**Affiliations:** Institute of Microbiology, University of Lausanne and University Hospital Center, Lausanne, Switzerland; Universitatsklinikum Hamburg-Eppendorf, GERMANY

## Abstract

Given the importance of microbiology results on patient care, high quality standards are expected. Internal quality assurance (IQA) could mitigate the limitations of internal quality control, competency assessment and external quality assurance, adding a longitudinal insight, including pre- and post-analytical steps. Here, we implemented an IQA program in our clinical microbiology facilities with blind resubmission of routine samples during 22 months. One-hundred-and-twenty-one out of 123 (98.4%) serological analyses and 112 out of 122 (91.8%) molecular analyses were concordant. Among the discordances in molecular biology analyses, 6 results were low positive samples that turned out negative, likely due to stochastic repartition of nucleic acids. Moreover, one identified retranscription error led us to implement automated results transmission from the Applied Biosystems instruments to the laboratory information system (LIS). Regarding Gram stain microscopy, 560 out of 745 (75.2%) of compared parameters were concordant. As many as 67 out of 84 (79.8%) pairs of culture results were similar, including 16 sterile pairs, 27 having identical identification or description and semi-quantification and 24 only showing variations in semi-quantification with identical description or identification of colonies. Seventeen pairs had diverging identification or description of colonies. Culture was twice only done for one member of the pairs. Regarding antibiotic susceptibility testing, a major discrepancy was observed in 5 out of 48 results (10.4%). In conclusion, serological tests were highly reproducible. Molecular diagnosis also revealed to be robust except when the amounts of nucleic acids present in the sample were close to the limits of detection. Conventional microbiology was less robust with major discrepancies reaching 39.5% of the samples for microscopy. Similarly, culture and antibiotic susceptibility testing were prone to discrepancies. This work was ground for reconsidering multiples aspects of our practices and demonstrates the importance of IQA to complete the other quality management procedures.

## Introduction

Microbiology laboratories are nowadays facing numerous challenges. The spread of antimicrobial drugs resistances demands rapid and yet reliable characterization of causative agents to prevent treatment failures and evitable occurrences of resistances [1–3]. Regular identification of emerging pathogens [3] and implementation of new molecular techniques [4] bring greater technical complexity, while prompt results and better cost-efficiency are expected [5–7]. Besides these evolutions, the awareness about medical errors and their consequences emphasizes the great importance of quality in health [8,9]. Indeed, clinical laboratories and related quality management programs have a role in preventing deaths due to evitable medical errors [10].

Quality management went along a constant evolution from the sixties to reach the current state of practice [11]. It is now generally accepted [12] and legally prescribed in Switzerland [13] and in the USA [14] that a minimal quality assurance (QA) program is composed of quality control (QC), external quality assessment (EQA), standard operating procedure (SOP) and competency assessment (CA) of coworkers. These procedures, despite being needed and regulated by certification processes such as ISO 15189 [15], have major limitations discussed below.

First, QC focuses on the intrinsic performances of laboratory tests, reviewing their performance (specificity & sensitivity), the regular maintenance of all used materials, the constant quality of reactants and media, as well as the use of negative and positives controls [12]. Despite their established role, QC are insufficient [11], especially because they do not cover pre- and post-analytical steps [10,16,17].

EQA includes external audits as well as proficiency testing (PT) with samples sent by reference nodal laboratories to be analyzed and described [12]. Audits can reveal structural dysfunctions and possible points to improve. PT with unknown samples probes the ability of the laboratory to treat and characterize samples and to identify pathogens. Both audits and PT provide valuable information on the functioning of the laboratories but are limited by their rare occurrence. PT has shown several additional limitations:

- It only partially evaluates pre- and post-analytical steps [18]; for example, PT samples are often coming in modified forms (e.g. dried, synthetically produced, pre-treated as with serum samples instead of whole blood), which can prevent their inclusion into regular lines of sample handling.
- PT are not available for all the different tests offered by our laboratories (for instance no PT is available for unusual pathogens such as *T*. *whipplei* and *W*. *chondrophila*) [19].
- It has been shown that their recognition as EQA can lead to falsely reassuring results because of the extra caution given in their processing [20,21]; indeed, samples labeled as EQA were shown to provide more reliable results than EQA submitted blindly [22].

Competency assessment (CA) probes the ability of the laboratory personnel to complete properly their tasks, in good adequacy with SOP. CA programs can be simple, such as direct observation of technicians and quizzes to complete, or more sophisticated, with for instance, submission of blind challenge samples [23–28]. Despite the different approaches evaluated in the literature, most suffer of similar limitations as QC, with a primary focus on individuals performing defined analytical phases and limited insight on the overall quality of laboratory processes. CA programs can also certainly be biased when the workers identify the time of evaluation. Interestingly, blind resubmissions of previously analyzed samples, which is one of the methods suggested in literature for CA purposes, seem to suffer of less limitations but are only seldom used [24].

Internal quality assurance (IQA) programs based on resubmissions of replicated samples could address the mentioned limitations but lack of supporting definitions and data. The potential of this type of procedures is recognized by reference organizations such as the Eurachem and the Standard Unit, Microbiology Services of Public Health England, which recommend them to assess internally the overall reproducibility of samples testing and handling [29,30]. However, there is very few original publications in clinical microbiology setting supporting these recommendations, the only ones being the studies conducted in Cambridge in the nineties by Constantine *et al* [31] and Gray *et al* [32]. The authors concluded then that their works showed the capacity of this system to detect error, highlighted point of improvements and produced relevant data on the reproducibility of their results and the overall quality of their laboratories [31–33].

Thus, in the present work, we implemented an internal quality assessment program using blind split samples. This provided data on the reproducibility of the results in our microbiology laboratory and helped us to define specific corrective measures.

## Materials and methods

### Study design

A new IQA procedure was initiated in October 2014 in the three clinical laboratories (serology, molecular microbiology and conventional microbiology) of the Institute of Microbiology of the University Hospital of Lausanne (Switzerland). This procedure was designed and implemented by the quality committee of our laboratories and exempted from formal ethic committee approval based on its regulation regarding quality control studies [34]. Indeed, the scheme which was applied here is in line with IQA procedure recommended by reference organizations in routine practice [29,30]. The present work reviews the data gathered over a 22-month period and evaluated the discrepancies, including their possible origin and their putative clinical impact (see below).

### Data collection

The head technicians of our three laboratories were instructed to secretly withdraw samples out of the already processed ones. In serology and molecular biology laboratories, samples were chosen to be proportionally representative of the analyses done by our facilities, but also ideally, to test at least once every test offered by our service. In the classical microbiology laboratory, the head technician generally selected samples that were expected to be challenging, because they were positive at Gram stain examination of by culture after overnight incubation. This approach was retained to increase the number of samples for which the adequacy of bacterial identification and antibiotics susceptibility could be investigated. The analysis request forms were replicated with anonymous identification data, which are commonly used in our institution for clinical research purposes (and thus not assimilated by our technician to a quality control). Part of the original sample material was taken into a new container and handed back in secret at the reception of the laboratories. The correspondence between the patient identification code and the replicate identification code was reported by the head technicians into a database for later comparison of the results. Results of the two matching samples were later reported into an Access database and data analyses were conducted with Microsoft Excel and PRISM (GraphPad Software). All data were processed anonymously.

### Serology and molecular biology

Pairs of serology results were compared for agreement between qualitative results (positive, indeterminate or negative) and variations in titers when relevant. Similarly, results of qPCR pairs were compared for differences in (i) qualitative results (positive versus negative), (ii) number of copies/ml with a margin of acceptance of one log_10_ between the two results and (iii) cycle thresholds (Ct) with a margin of acceptance of 3.3 Ct between the two results.

### Gram stain examination

Gram stains results were compared regarding semi-quantification of the following parameters: leukocytes, erythrocytes, epithelial cells, yeasts, Gram-positive cocci, bacilli and diplococci and Gram-negative cocci, bacilli and coccobacilli. The given interpretations of the Gram results were also compared when relevant (e.g. suspicion of urines contamination, salivary flora in sputa, dominant bacterial flora in vaginal swabs). For each parameter, the amplitude of discrepancies was classified according to S1A Table. The most important discrepancy between all parameters of a pair of samples was recorded. We examined the discrepancies for each parameter by linearly weighted Kappa score using GraphPad QuickCalcs website [35]. Kappa score quantifies the level of correspondence between the ratings of two observers, considering the natural probability to obtain the same level of concordance only by chance [36]. The weighted version of Kappa score is used to consider the partial agreements within ordered scales [37]. We adopted the weighting suggested by Cicchetti et Allison [38], which sanctions discrepancies in a linear and not in a quadratic method [39].

### Culture and antibiotic susceptibility testing (AST)

Culture results were compared for description and/or identification and semi-quantification and discrepant cases were described. Quantitative variations were scored according to S1B Table.

Sets of AST were compared when conducted for the same identified bacteria within a pair of replicates. Discrepancies were classified following S1C Table. The most significant discrepancy within all tested antibiotics was retained to grade the concordance of the whole AST sets (e.g. if the AST pairs included a single major discrepancy, the pair of sets were considered as being majorly discrepant). If AST were not performed for the same species on both replicates, the cause of the discrepancy was analyzed and described.

### Evaluation of clinical impact and putative cause

Interpretation of the clinical impact and putative cause of discordant results was done by a data review committee of three persons (VS, CD and GG). Discrepancies were classified as clinically negligible or significant.

## Results

### Serology

Among 123 serological tests performed twice on 35 sera, a total of 121 (98.4%) were concordant (Table 1). The first discordant test was a measure of IgG against cytomegalovirus (CMV) by enzyme immuno-assay (EIA), which was initially positive (index of 1.20) and intermediate when redone (0.953 for a cut-off for positivity of 1 and for negativity of 0.5). The 123 tests were often completed within panels of multiple tests detecting antibody reactivity for the same pathogen (in total 76 different pathogen-specific panels), sometimes reducing the negative impact of discordant results. Thus, this first discordant result was accompanied by electrochemiluminescence (ECLIA) results, which were both times positive for anti-CMV IgG antibodies (4.84 and 4.78 U/ml with a cutoff of 1 for positivity). The second discordant pair of results was in the titers of a syphilis test (TPPA) obtained manually, being twice positive but with titers of 1/2'560 initially and 1/10’240 when redone. The clinical impact was considered significant in this 2^nd^ case since a high titer of 1/10’240 generally reflects an acute infection and since this difference of two dilutions might be considered as clinically relevant [40,41]. The limited number of discrepant results did not allow to investigate the effect of the time delays between the management of the original samples and their replicates.

**Table 1:** Overview of results agreements in our different laboratories.

	Percentage of agreement	Discordant analyses	Clinically significant discordances	Time between replicates
				Mean (SD)
				p25/p50/p75 (in hours)
**Serology**	98.4%	2/123	1/2	28 (19.1)
				19.1/24.6/41.6
**Molecular diagnosis**	91.8%	10/122	6/10	
Qualitative results	95.1%	6/122	6/6	182.8 (45.8)
Quantitative results	82.6%	4/23	0/4	169.1/191.5/215.8
**Microscopy**	75.2%*^1^	185/745	13/71*^2^	10.3 (9.5)
**Culture**	50.0%	43/86	13/43	
**AST**				
Aggregated by panels	75.0%	12/48	1/12	5.0/6.5/8.2
Single antibiotics	96.4%	25/759	1/25	

This table shows an overview of the agreement, discordance and clinical significance of discordances for the three laboratories. The column “*Time between replicates*” indicates the distribution of time, in hours, between the management of the original sample and its replicate. For AST, results were analyzed once to see whether the panels done for the same bacteria had identical results (*Aggregated by panels*) and once to see whether every single antibiotic tested had the same results twice (*Single antibiotics*).

*^1^ overall agreement for all parameters;

*^2^ results aggregated per samples;

SD = standard deviation; p25 = percentile 25; p50 = percentile 50, p75 = percentile 75.

### Molecular diagnosis

Among 122 tests achieved on 52 replicated samples, 112 (91.8%) molecular tests were concordant (Table 1). Six tests were qualitatively different, being once positive and once negative. Of the 23 results pairs that included quantitative results, three were diverging in the number of copies/ml (difference in copies/ml over one log_10_) and one was quantitatively different only considering the Ct values (difference in Ct over 3.3).

**Fig 1:**
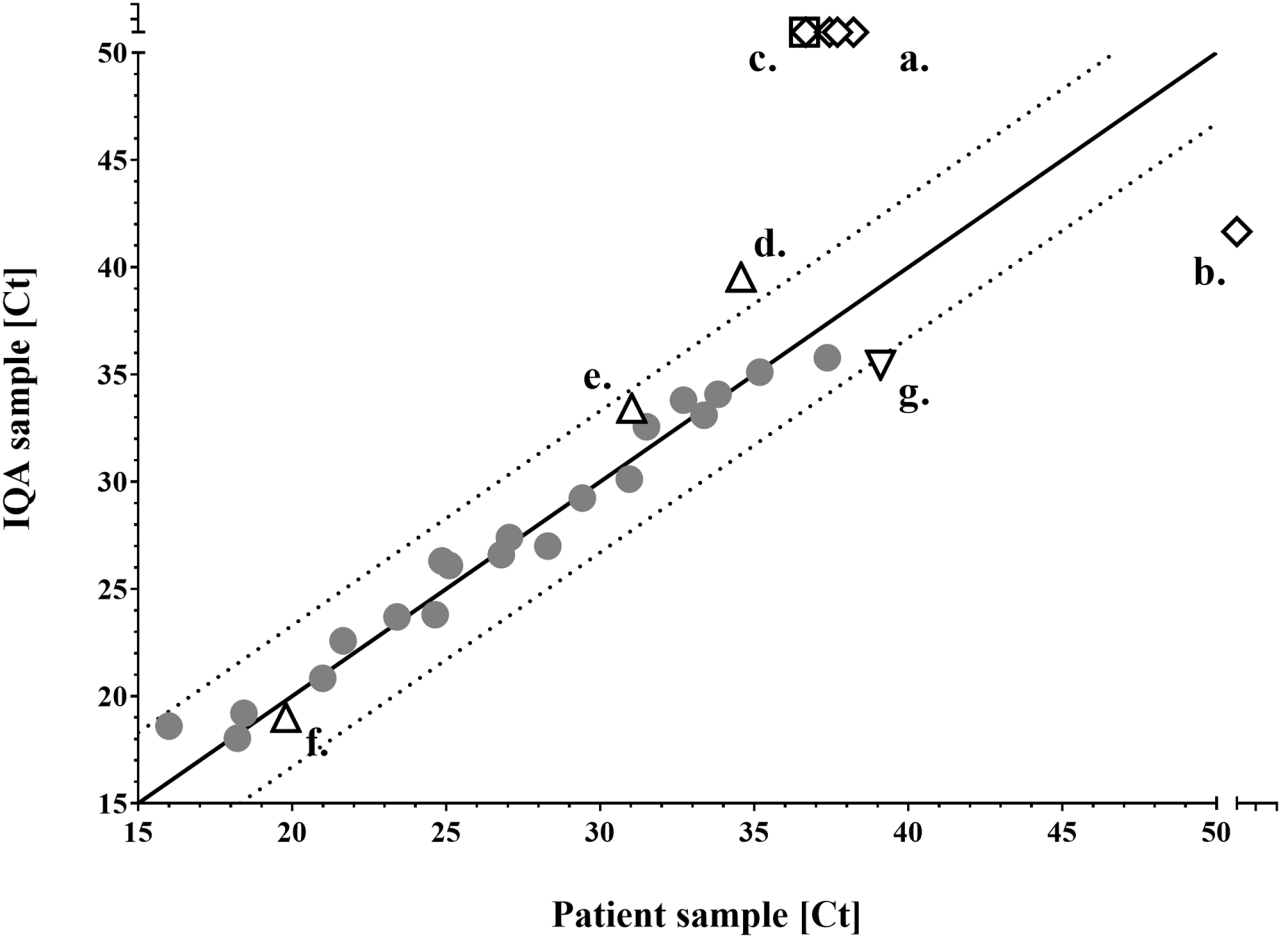
Molecular biology results pairs reparation. Straight line represents the line of identity, expressing perfect match. Pointed lines delaminate a ± 3.3 Ct tolerance margin. Represented are low positive versus negative results (n = 5, diamonds); moderate positive versus negative results (n = 1, square), quantitative discrepancies with over one log_10_ difference in copies/ml (straight triangles, n = 3), quantitative discrepancy with difference over 3.3Ct but less than one log10 (reversed triangle, n = 1); agreeing positive results (rounds, n = 20). 92 negative agreeing results are not represented on this figure. Note that these results here include two negative pairs and one positive pair of tests that were not quantitatively reported in copies/ml but only reported in Ct number by the laboratory.

Regarding the qualitative differences, four discordant molecular results corresponded to low positive samples (132–175 copies/ml) that turned out to be negative when retested (Fig 1a) whereas one initially negative result turned out to be positive at 100 copies/ml when retested (Fig 1b). These 5 discordant results corresponding to EBV in blood (n = 3) and BKV in blood and urine (n = 2) DNA amplifications could be explained by stochastic repartition of nucleic acids in the samples. The remaining qualitatively discordant result corresponded to the detection of a coronavirus in a nasopharyngeal secretion, initially positive at 2280 copies/ml with a Ct of 36.6, and negative when redone (Fig 1c). Even though all these six qualitative discrepancies were expected since being below the level of sensitivity of our PCRs, we considered that such results may have a significantly different clinical impact (Table 1). The analysis conducted on the effect of time on the discrepancy level was inconclusive because of the small number of discordant results. The average time between original testing and replication was longer for concordant results than for discrepant.

Concerning the four quantitative discordances out of the 23 samples pairs that included quantitative results, a *Bordetella pertussis* detection in a nasopharyngeal secretion was discordant both considering the bacterial load (2700 copies/ml versus 260 copies/ml) and the Ct values (34.6 versus 39.5) (Fig 1d). However, this difference was not considered clinically significant (Table 1). A Parainfluenza 2 also tested in a nasopharyngeal secretion was initially reported at 505’000 copies/ml and then reported at only 50’000 copies/ml when redone, a difference not considered to be associated with a significant clinical impact; this one log_10_ difference in viral load quantification was likely due to a technical error when redone since the CT difference was of 2.4 (31.0 versus 33.4) (Fig 1e and Table 1). The third quantitatively discordant result corresponded to the detection of HSV-1 in a vaginal swab with a viral load of 9.6×10^10^ copies/ml versus 1.6×10^8^ copies/ml, a difference identified to be due to a human transcription error since the Ct values were similar (19.8 and 19.0) (Fig 1f). This error was sufficient to lead to a change of process with implementation of automated data transfer from the PCR automated system to the Laboratory Information System (LIS), but was not considered to have a significant clinical impact (Table 1). The fourth quantitative discordance had little clinical impact (Table 1) despite the Ct difference (39.1 versus 35.4) since the calculated EBV viral load exhibited a difference of less than one log_10_ (182 versus 771 copies/ml) (Fig 1g).

### Conventional microbiology

Ninety-seven samples were duplicated and resubmitted to the conventional microbiology laboratory, requiring variable combinations of Gram staining, culture and AST. Four samples pairs results were excluded of our analysis. Two because they were including only parasites in stool analyses, too few to be considered; one because it was possibly being subject to an inversion in IQA sample preparation since staining and cultures results were incompatible; one because the request for analysis forms were not demanding for the same analyses.

Regarding microscopy of the Gram stains, 32 out of 81 (39.5%) pairs exhibited at least one major discrepancy (Tables 1 and 2), mainly due to discordances in the reported quantities of red blood cells (19.8%), leukocytes (8.6%) and Gram-positive cocci (8.6%) (Table 2). Major discordances were observed in 5.3%, 4.9% and 3.7% for Gram-negative coccobacilli, Gram-negative bacilli and Gram-positive bacilli, respectively (Table 2). Overall agreement of the microscopy was of 75.2% (560/745) of all evaluated parameters, whereas the occurrence of a major error occurred in 6.4% of cases (48/745) (Tables 1 and 2). However, this relatively high overall agreement rate should be weighted by the common negativity of some parameters such as Gram-positive diplococci (100% of negativity), yeasts (96.3%) and Gram-negative coccobacilli (93.0%). Thus, after excluding those parameters, the obtained overall agreement would drop significantly from 75.2% to 67.6%. True rate of concordance is better assessed by weighted Kappa scores, shown in Table 2 for each parameter. As many as 13 out of 71 (18.3%) samples exhibiting at least one discrepancy were considered as having a clinically relevant discordance, corresponding to a rate of 16.0% (13/81) for all Gram stain results investigated (Table 1). The discrepancies were considered by our experts to be possibly related to reading or reporting errors in 8 out of 13 significant and in 11 out of 58 negligible discrepant pairs of samples, while the rest seemed compatible with natural biological variations (e.g. sample and coloration heterogeneity). It should also be noted that for two samples, the Gram stain reading was done only for one of the replicates because of pre-analytical disagreement on the aspect of urine (urines are examined under microscopy only if considered cloudy). Regarding the effect of the time delay between the management of the original samples and their replicates, no significant differences were observed, when comparing the identical and negligible discrepant results versus the minor and major discrepant results. This absence of difference was recovered both when comparing all parameters together and bacteria and yeasts only.

**Table 2:** Repartition of Gram staining discrepancies.

	Identical result		Negligible discrepancy		Minor discrepancy		Major discrepancy		Total	Rate of double negativity	Weighted Kappa
**Leukocytes**	37	*45*.*7%*	29	*35*.*8%*	8	*9*.*9%*	7	*8*.*6%*	81	*2*.*5%*	0.49
**Erythrocytes**	51	*63*.*0%*	9	*11*.*1%*	5	*6*.*2%*	16	*19*.*8%*	81	*33*.*3%*	0.55
**Epithelial cells**	58	*71*.*6%*	11	*13*.*6%*	6	*7*.*4%*	6	*7*.*4%*	81	*60*.*5%*	0.62
**GP cocci**	49	*60*.*5%*	21	*25*.*9%*	4	*4*.*9%*	7	*8*.*6%*	81	*46*.*9%*	0.66
**GP diplococci**	57	*100*.*0%*	0	*0*.*0%*	0	*0*.*0%*	0	*0*.*0%*	57	*100*.*0%*	N.R.
**GN cocci**	60	*93*.*8%*	3	*4*.*7%*	0	*0*.*0%*	1	*1*.*6%*	64	*92*.*2%*	0.69
**GN bacilli**	48	*59*.*3%*	18	*22*.*2%*	11	*13*.*6%*	4	*4*.*9%*	81	*49*.*4%*	0.63
**GN coccobacilli**	53	*93*.*0%*	1	*1*.*8%*	0	*0*.*0%*	3	*5*.*3%*	57	*93*.*0%*	N.R.
**GP bacilli**	69	*85*.*2%*	8	*9*.*9%*	1	*1*.*2%*	3	*3*.*7%*	81	*70*.*4%*	0.79
**Yeasts**	78	*96*.*3%*	2	*2*.*5%*	0	*0*.*0%*	1	*1*.*2%*	81	*96*.*3%*	N.R.
**Overall**	560	*75*.*2%*	102	*13*.*7%*	35	*4*.*7%*	48	*6*.*4%*	745		
**Highest discrepancy**	**10**	***12.3%***	**19**	***23.5%***	**20**	***24.7%***	**32**	***39.5%***	**81**		

Gram staining of routine samples and IQA samples were compared and discrepancy amplitudes were categorized according to S1B Table for each parameter. Rate of double negativity represents the proportion of results that were negative for both the routine and IQA sample. Weighted Kappa score express the level of concordance between two rates, not only due to natural probability of agreement. Score of -1 expresses perfect disagreement; 0 expresses agreement corresponding to random; probability; 1 expresses perfect agreement. [36,37]

*GP* = Gram-positive; *GN* = Gram-negative; NR = non-relevant

Culture results were perfectly identical for 43 pairs of samples, 16 of which were twice sterile (Tables 1 and 3). For 24 other pairs of samples, the results showed only negligible or minor discrepancies within semi-quantitative scoring of similarly identified or described colonies, whereas 17 pairs showed discrepancies within the identification or description of colonies, as detailed in Table 3. To be noted, two pairs of samples for which cultures were accomplished only for one member of the pair: once due to diverging microscopy results with a potentially significant impact and once to an error in procedure with negligible impact (Table 3). Also noteworthy, three cases in which the differences in microscopy results motivated the additional inoculation of Gram-positive selective agar, explaining why supplementary clinically-significant strains (*Enterococcus faecalis* or *Staphylococcus aureus*) were recovered from cultures (Table 3). Interestingly, when comparing the time delay from original samples processing to management of the replicates, the delay was significantly longer for samples that included minor or major discrepancies in culture growth versus the ones that were identical or negligibly discrepant (14.68 hours ± 2.45 versus 9.19 hours ± 1.13, p = 0.039).

**Table 3:** Culture results: Agreement and description of discrepant cases.

**No culture achieved for both replicates**	**7**
**Culture achieved only for one replicate**	**2**
Unnecessary cultivation of vaginal swab for the IQA sample, with otherwise agreeing microscopy results showing no potential pathogen. Difference of procedure considered as of negligible impact.	
Respiratory sample was not cultivated for patient’s sample because of microscopy results suggestive of a contamination, whereas the IQA microcopy results led to cultivation and growth of moderate quantities of ***Klebsiella oxytoca*** * *and* ***Escherichia coli*** * along a physiological mixed oropharyngeal flora. Difference of procedure considered of significant impact.	
**Completely identical identification or description and semi-quantification**	**43**
* Including completely sterile pairs of culture*	*16*
**Identical identification or description, negligible variation in semi-quantification**	**24**
**Culture presenting discrepant identification or description**	**17**
* Negligible discrepancies*	*5*
Low quantities of ***C. albicans*** recovered only in the patient’s respiratory sample but not in the IQA replicate, with otherwise agreeing results (***Staphylococcus aureus*** and oropharyngeal flora)	
Low quantities of ***C. albicans*** recovered only in the patient’s respiratory sample, with otherwise agreeing results (***Escherichia coli***, yeast other than ***C. albicans***)	
Low quantities of ***Pseudomonas fluorescens*** * recovered only in the patient’s pharyngeal swab sample, with otherwise agreeing results (oropharyngeal flora)	
***Bifidobacterium*** spp (10^7^ bacteria/ml) recovered only in the patient’s urinary sample, with otherwise agreeing results (***Enterococcus faecalis***)	
Two strains of ***Staphylococcus aureus*** * detected in the respiratory IQA sample, whereas only one strain was reported in the patient’s sample, with diverging antibiotic susceptibility between the two strains only for penicillin	
* Significant discrepancies*	*12*
Similar growth on plates but different interpretation with only a description of the grown colonies for the patient’s respiratory sample (low quantities of ***Enterobacteriaceae***, 3 different morphologies and oropharyngeal flora); this mixed bacterial flora turned out to include low quantities of ***Klebsiella pneumoniae*** * and of ***Enterobacter cloacae*** * group bacteria, with a physiological oropharyngeal flora, when the Gram-negative colonies were properly identified for the IQA sample	
Similar growth but different interpretation with only a partial identification for the patient’s respiratory sample (low quantifies of ***Enterobacter*** **cloacae** group and 3 other morphologies) which turned out to include low quantities of ***Serratia marcescens*** * when properly identified for the IQA sample	
Similar growth but different interpretation with only description for IQA respiratory sample (low quantities of Gram-negative ***Enterobacteriaceae*** with oropharyngeal flora) which turned out to include low quantities of ***Escherichia coli*** * when properly identified for the patient’s sample. This difference of procedure could be the consequence of differences in the Gram stain results (slightly more abundant leucocytes and Gram-negative cocci may have motivated formal identification for the patient’s sample)	
Low quantities of ***Escherichia coli*** * and moderate quantities of ***Enterococcus faecalis*** * recovered only in IQA ascites liquid sample, while initial patient sample was sterile. The Enterococcus grew on a Gram-positive selective plate only used for the IQA sample because of abundant Gram-positive cocci seen under microscopy. Possible error in samples handling, during the preparation of the IQA sample, since Gram results were also discordant	
Low quantities of ***Escherichia coli*** * recovered only in IQA ascites liquid sample, while patient’s sample stayed sterile	
***Escherichia coli*** * (10^7^ germs/ml) and ***Enterococcus faecalis*** (10^7^ germs/ml) recovered only in patient’s urinary sample, while IQA sample stayed sterile. Suspicion of error in sample handling	
***Enterococcus faecalis*** * (10^5^ germs/ml) recovered only in IQA urinary sample, in otherwise agreeing results (***Proteus vulgaris***). This difference was explained by the usage of a Gram-positive selective agar plate since abundant Gram-positive cocci were observed under microscopy for IQA sample	
Moderate quantities of ***Staphylococcus aureus*** * recovered only in the patient’s sample (ear swab), with otherwise agreeing results (***Pseudomonas aeruginosa***). This difference was explained by the usage of Gram-positive selective agar plate since abundant Gram-positive cocci were observed under microscopy for the patient’s sample	
Low quantities of ***Escherichia coli*** * recovered only in the patient’s respiratory sample, in otherwise agreeing results (***Enterobacter*** **cloacae** group and ***Pseudomonas aeruginosa***)	
Low quantities of ***Klebsiella*** **oxytoca** group, ***Escherichia coli*** and ***C. albicans*** recovered only in the patient’s sample (a biliary liquid), with otherwise agreeing results (***Enterococcus faecium, Ochrobactrum*** spp).	
Low quantities of ***Streptococcus*** **anginosus/milleri** group recovered only in the IQA sample (a superficial wound swab), with otherwise agreeing results (***Staphylococcus aureus***)	
High quantities of ***Bacteroide*s fragilis** group recovered only in the patient’s urine sample, with otherwise agreeing results (***Escherichia coli*** and ***Enterococcus faecalis***)	
**Total**	**93**

* indicates the colonies that led to an AST when present.

Regarding AST panels, at least one major discrepancy was observed in five (10.4%) out of 48 comparable AST panels, whereas 7 (14.6%) included at least one minor discrepancy, the remaining 36 AST panels (75.0%) being perfectly identical (Table 4). These discordances had little clinical impact since only one minor discrepancy concerned an antibiotic which would potentially have been used in this situation (a *Pseudomonas aeruginosa* in a respiratory sample which was once susceptible and once intermediate to levofloxacin) (Table 1).

**Table 4:** Comparison of AST panels accomplished for the same identified bacterial species.

Identical AST results	36	*(75.0%)*
AST including at least one minor discrepancy	7	*(14.6%)*
AST including at least one major discrepancy	5	*(10.4%)*
**Total**	**48**	

Major and minor categories as defined in S1C Table.

As many as 23 AST panels, done on the patient sample or the IQA replicate, had no matching AST panel for its counterpart, either because of different interpretation of similar culture results (n = 8) or because of different culture results (n = 15) (Tables 3 and 5). The clinical significance of the 8 AST panels not performed in presence of identical culture results is negligible, especially since 5 of them were accompanied in transmitted results by a comment suggesting to the clinician to ask for AST via telephone if needed. Nevertheless, this difference in attitude could possibly influence the clinical interpretation by unexperimented practitioners. On the contrary, 14 out of 15 of the identified bacteria that led to diverging presence of AST were considered as significant (species marked by * in Tables 3 and 5)

**Table 5:** AST panels without panels for the same bacteria in the results of the corresponding sample.

**Identical culture results, different interpretation**	**8**
* Accompanied by a comment suggesting a request for AST via phone call*	*5*
* Not accompanied by a comment*	*3*
**Diverging culture results, leading to different AST**	**15**
* Strain presence discrepancy considered as negligible in culture* (Table 3)	*1*
* Strain presence discrepancy considered as significant in culture* (Table 3)	*14*
**Total**	**23**

**Fig 2:**
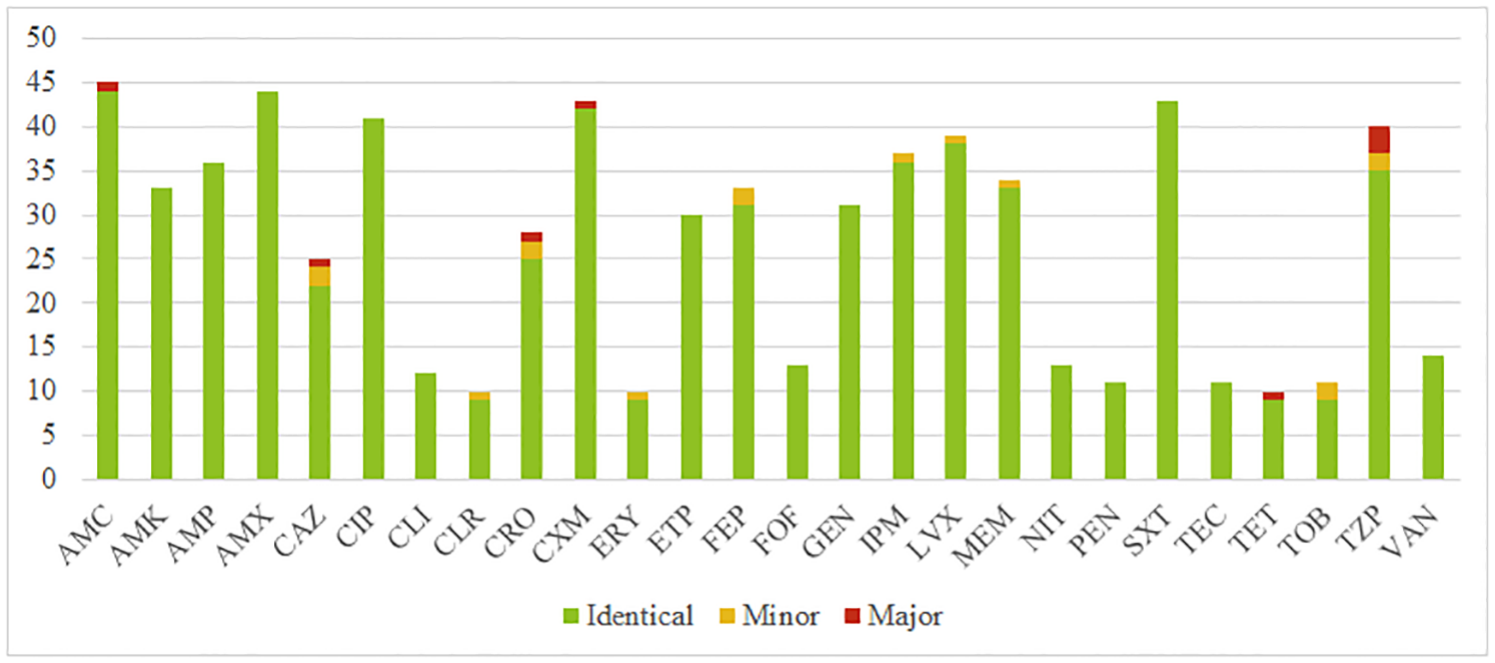
AST agreement, per antibiotics. Counts of concordant pairs of AST results are in green, minor discrepancies (sensitive versus intermediate or intermediate versus resistant) are in yellow and major discrepancies (sensitive versus resistant) are in red. Only antibiotics tested more than 10 times are represented. Complete results are available in S2 Table. AMC = amoxicillin-clavulanic acid; AMK = amikacin; AMP = ampicillin; AMX = amoxicillin; CAZ = ceftazidime; CIP = ciprofloxacin; CLI = clindamycin; CLR = clarithromycin; CRO = ceftriaxone; CXM = cefuroxime; ERY = erythromycin; ETP = ertapenem; FEP = cefepime; FOF = fosfomycin; GEN = gentamicin; IPM = imipenem; LVX = levofloxacin; MEM = meropenem; NIT = nitrofurantoin; PEN = penicillin; SXT = trimethoprim-sulfamethoxazole; TEC = teicoplanin; TET = tetracycline; TOB = tobramycin; TZP = piperacillin-tazobactam; VAN = vancomycin.

When AST were compared individually, each tested antibiotic one by one, the overall agreement was as high as 96.7% (734/759). There were 15 minor (sensitive versus intermediate or intermediate versus resistant) and 10 major (sensitive versus resistant) discrepancies. Piperacillin-tazobactam was the antibiotic the most often discrepant, with 3 major and 2 minor discrepancies. Detailed analysis of AST results, ordered by antibiotics, is shown in Fig 2, which represents concordances and discordances for antibiotics tested at least 10 times. All other results are available in S2 Table.

Nine pairs of sample included rapid testing such as tests for *C*. *difficile* antigens and inducible beta-lactamase. All these tests were concordant.

### Mycobacteria

Five pairs of samples submitted to conventional microbiology or molecular biology laboratories also included auramine stains, culture and AST for mycobacteria. All the results were concordant, showing agreement on two positive samples and three negative samples.

## Discussion

This work demonstrates the feasibility and usefulness of an internal quality assurance program as part of the global quality management in serology, molecular biology and conventional microbiology laboratories. Looking at the level of agreement of the different laboratories, we first observe the high robustness of serological tests, which is partially due to automated systems and commercial kits which are mostly used, enhancing the reproducibility of processes. Moreover, serological tests are achieved on sera, which are by nature homogenous, limiting the variability between different fractions of a given sample.

In molecular biology, the usage of IQA samples shows the common occurrence of discrepancies (10/122 analyses, Fig 1). Most discrepant results were due to the stochastic variability of qPCR occurring around the limit of detection, where by definition qPCRs lose part of their sensitivity [42]. Accordingly, these discrepancies should not be considered as technical errors or insufficiencies. Yet, this variability for weakly positive samples may be overlooked by clinicians who often give too much importance to negative results, even when the negative predictive value is known to be low (i.e. for *M*. *tuberculosis* detection and for *Listeria* PCR in cerebrospinal fluid). While such discrepancies are of limited implication in terms of quality assurance, the single quantitative discrepancy of a 610-folds factor between copies/ml, with almost identical Ct number, was worrisome. Investigations revealed that this difference was the consequence of an error in the manual transcription of the result. In this specific situation, this error did not have clinical impact, since both results would be interpreted as highly positive by clinicians. However, this error highlighted a weak point in our procedures and led us to integrate our automated PCR system with a middleware capable of two directions data transfers from the Applied Biosystems PCR machine to the LIS, in order to definitively abolish any risk of human errors during results retranscription. Made worthy, no false positive seem to have resulted from PCR contaminations. This is particularly remarkable considering the high sensitivity of PCR and likely results from the advanced automatization of our qPCR platform [19]. Moreover, our IQA process proved itself useful to detect potential errors in this laboratory.

Gram stain reading is well established as a complex procedure that has number of pitfalls and its poor reliability has been described at multiple occasions [43–47]. It is still worrisome to observe that almost 40% of the compared results included at least one discrepancy of more than two relative units, which we categorized as major (Table 2). This rate cannot be directly compared to any other data available in literature, the only similar experiment being the study of Constantine et al. (14), which had only 2% of discrepancies for microscopy, but using a very different definition of discrepancy which allowed much larger differences in the semi-quantitative results. The other studies on the reproducibility of Gram stain use very different methodologies, such as comparison of the concordance between Gram stain and culture results or between different observers reading identical smears, thus providing non-comparable results [44–47].

Observing the results by parameters in Table 2, the repartition of reported results should be considered. For example, the very high Gram-positive diplococci or yeasts concordance is associated with a very high level of negativity. Kappa score partially helps to compare parameters between them, taking into account the natural proportion of concordance explained by random probability [36]. According to this score, we conclude that in our laboratory the counts of host cells (leukocytes, erythrocytes) is less reliable than the counts of bacteria. This questions the sense of reporting parameters such as erythrocytes, with such a high level of variability and low clinical impact. Those data also support the need for continuous staff training in this domain and the integration of automatization in Gram reading to improve reproducibility.

In the evaluation of error rates in Gram staining examination, it is important to emphasize that our study included no gold-standard. Thus, our experts (GGR and CDU) could only subjectively assess the most likely origin of the discrepancies. Errors were typically suspected when discrepancies were not compatible with biological variations because they were of very large amplitude or when confusion was suspected (e.g. ++++ Gram-positive cocci versus ++++ erythrocytes). Nevertheless, the factors leading to result heterogeneity and errors are numerous for Gram stains. Indeed, the important heterogeneity of samples such as sputa or fragments and the heterogeneity induced by the staining process can already bring a lot of variability before the reading phase itself, which is a task known as subjective and complex. Moreover, despite the high proportion of discrepant cases, our experts concluded that most of them would have no clinical impact, mainly because they involved host cell quantifications and not detection of bacteria (Table 1). Furthermore, the clinical impact of Gram stains itself is subject to discussion, as for the diagnosis of pneumonia [48,49]. Nevertheless, if the importance of Gram results can be relativized, our work also showed that discrepancies in microscopy results can influence subsequent analytical steps, with significant impact on final culture and AST results transmitted to the clinicians (Tables 3 and 5).

Reviewing the comments transmitted with the Gram stain results, an interesting observation was made concerning 4 out of 22 urinary samples that had contradictory comments which concluded once to negativity and once to positivity for the corresponding replicate. These discrepancies were caused by relative quantifications of bacteria that were scored “few” for one sample and “+” or “++” for the other, with an automatic comment announcing positivity in the second case only. Comparing those results with the culture results performed on the same samples, all 4 samples were confirmed positive. This observation questions the usefulness of the “few” category.

The results of culture shown in Table 3, with 12 significant differences out of 84 cultures pairs (85.7% accuracy), are representative of its complexity. Culture is a dynamic process influenced by many factors, including heterogeneity of samples, Gram stains results, selected culture media, stochastic growth variability and interpretation of the clinical significance of the presence of colonies with choices to identify or not the grown colonies. All these factors contribute to explain the observed discrepancies. Better homogenization of the samples, reconsideration of how Gram results are used to select culture media and training of the personnel concerning analytical choices are all possible ways to improve reliability of the culture steps. Furthermore, regarding the design of an IQA program, longer time between the treatment of the original samples and their replicates was in our data associated with higher level of discrepancy in cultures results. Even this is to be expected, it underlines the importance of shortening the time delay between the inoculation of the original samples and the replicates.

Interestingly, we observed a low rate of significant discrepancies within the AST performed on identical bacteria (Table 4, Fig 2 and S2 Table). On the other hand, steps preceding accomplishment of AST (Gram staining, cultivation, identification and interpretation of culture) often influence significantly the AST transmitted to the clinicians (Tables 3 and 5). These observations make us consider that the accuracy of the analytical phase of AST is satisfactory and that we should focus on the pre-analytical factors that are influencing their realization. It could be argued that the clinical information available concerning the IQA samples were different (e.g. the age) or lacking (e.g. the service of hospitalization), influencing the interpretation of the culture. If this may explain some discrepancies in the interpretation of similar culture results, discrepancies originating from diverging interpretation of similar cultures concerned only a minority of samples (Table 5).

Our study, which included only a limited number of samples, provided interesting data on reproducibility. However, samples were not totally chosen in blind but sometimes selected because they addressed a particular analytical question. For instance, cloudy urinary samples were preferably chosen over limpid samples and positive samples are certainly over-represented, increasing the probability of diverging results.

Another limitation of our IQA program concerns specifically the serology laboratory. First, in this facility, blood samples are coming in gel-based serum separating tubes. They are centrifuged and the supernatant is directly transferred into new tubes. This implies that the replicates could only come from already treated samples and that their visual aspect was different from almost all routine samples. The technicians of this facility are routinely checking anterior results from the tested patients to integrate the new results within their serological status. Because we were using a limited pool of anonymous patient profiles, our technicians eventually faced multiple anterior results corresponding to IQA samples processed earlier. Both the visual difference of samples and the anterior results from anonymous patient’s profile led to strong suspicions of the staff working in the serology laboratory about our ongoing experimental IQA process, which finally obliged us to discontinue the IQA in this laboratory. This specific issue did not concern the two other facilities where our process could be completed in secret and on samples that were not pretreated. Despite this limitation, we consider that our process could still have an interest for IQA in serology laboratories, but that it would need to be partly redesigned. A proposition would be to duplicate samples before their arrival in the laboratory. It would solve the problem of visual impact and improve the coverage of pre-analytical steps. It would also be necessary to use more sophisticated fake patient identities, without previous IQA results to prevent their recognition by our coworkers.

Finally, even if our results cover more steps than traditional quality processes, it is still not covering all steps from prescription to interpretation of results by clinicians. Thus, we intend to extend this quality assurance project by collaborating with three other Swiss hospital laboratories. Each laboratory would challenge the others with split samples sent into containers accompanied by etiquettes and identifications used by the receiving laboratories for their external demands. This way, each laboratory would play the role of reference laboratory for one fourth of all samples. This approach would resolve the limitations of our scheme concerning the pre-treatment of the serology samples, the recognition of the samples and the lack of reference results. Such interlaboratory shared quality assurance would also help to provide “external assurance” for some serological and molecular parameters for which there is currently no EQA available (i.e. *Tropheryma whipplei*).

In conclusion, this study revealed insightful points to improve and questioned our current practices, such as the use of Gram results to select culture media. Also, this study confirms the great interest of an IQA scheme based on split samples reprocessed in blind. It represents only few hundreds supplementary analyses, on the over 25’000 analyses run every year in our laboratory and this allowed us to generate interesting data on our reproducibility. This design showed itself to be perfectly complementary to other quality management procedures already ongoing in our laboratory and it should be further developed to better delineate the identified limitations and to settle its place in the overall quality assurance program.

## Notes

Part of this publication was the subject of a master thesis done by Valentin Scherz under the direct supervision of Professor Greub at the School of Medicine of the University of Lausanne. Christian Durussel is the senior laboratory technician, acting at the Institute of Microbiology as the quality officer.

## Supporting information

S1 Table Categories defining amplitude of discrepancies in conventional microbiology laboratory. Categories defining the amplitude of the discrepancies observed between A. semi-quantification of parameters pairs of Gram reading; B. semi-quantification of grown colonies; C. Antibiotic susceptibility testing (AST) all performed twice, first as part of the routine diagnostic procedure and then as part of the IQA program.
(DOCX)

S2 Table Antibiotics sensitivity testing agreement, per antibiotics. Complete results of per antibiotics analysis of antibiotics sensitivity testing concordances and discrepancies. Minor discrepancies are either sensitive versus intermediate or intermediate versus resistant. Major discrepancies are sensitive versus resistant.
(DOCX)
