# Supplementary material for: Internal quality assurance in diagnostic microbiology: A simple approach for insightful data
Source: PLoS One. 2017 Nov 14;12(11):e0187263. doi: 10.1371/journal.pone.0187263 (PMC5685576; doi:10.1371/journal.pone.0187263)
Supplement: S1 Table — Categories defining the amplitude of the discrepancies observed between A. semi-quantification of parameters pairs of Gram reading; B. semi-quantification of grown colonies; C. Antibiotic susceptibility testing (AST) all performed twice, first as part of the routine diagnostic procedure and then as part of the IQA program. (DOCX) [file pone.0187263.s001.docx]

**S1 Table. Categories defining amplitude of discrepancies in conventional microbiology laboratory.**

|  |  | Negligible discordancy | Minor discordancy | Major discordancy |
| --- | --- | --- | --- | --- |
|  | **Gram stain** | ++++<->+++  +++<->++  ++<->+  +<->few  few<->0 | ++++<->++  +++<->+  ++<->few  +<->0 | ++++<->+  ++++<->few  ++++<->0  +++<->few  +++<->0  ++<->0 |
|  | **Culture** | strong <-> Moderate  moderate <-> low  x10^1^ difference | strong <-> low low <-> sterile  x10^2^ difference | sterile <-> moderate  sterile <-> strong  >x10^3^ x difference |
|  | **AST** |  | resistant <-> intermediate  sensitive <-> intermediate | resistant <-> sensitive |
