# Supplementary material for: Internal quality assurance in diagnostic microbiology: A simple approach for insightful data
Source: PLoS One. 2017 Nov 14;12(11):e0187263. doi: 10.1371/journal.pone.0187263 (PMC5685576; doi:10.1371/journal.pone.0187263)
Supplement: S2 Table — Complete results of per antibiotics analysis of antibiotics sensitivity testing concordances and discrepancies. Minor discrepancies are either sensitive versus intermediate or intermediate versus resistant. Major discrepancies are sensitive versus resistant. (DOCX) [file pone.0187263.s002.docx]

**S2 Table. Antibiotics sensitivity testing agreement, per antibiotics.**

| Antibiotics | Identical | Minor | Major | Total |
| --- | --- | --- | --- | --- |
| Amikacin | 33 | 0 | 0 | 33 |
| Amoxicillin | 44 | 0 | 0 | 44 |
| Amoxicillin-clavulanic acid | 44 | 0 | 1 | 45 |
| Ampicillin | 36 | 0 | 0 | 36 |
| Aztreonam | 2 | 0 | 0 | 2 |
| Cefaclor | 2 | 0 | 0 | 2 |
| Cefazolin | 7 | 0 | 0 | 7 |
| Cefepime | 31 | 2 | 0 | 33 |
| Cefoxitin | 1 | 0 | 0 | 1 |
| Cefpodoxime | 3 | 0 | 1 | 4 |
| Ceftazidime | 22 | 2 | 1 | 25 |
| Ceftriaxone | 25 | 2 | 1 | 28 |
| Cefuroxime | 42 | 0 | 1 | 43 |
| Ciprofloxacin | 41 | 0 | 0 | 41 |
| Clarithromycin | 9 | 1 | 0 | 10 |
| Clindamycin | 12 | 0 | 0 | 12 |
| Colistin | 3 | 0 | 0 | 3 |
| Doxycycline | 7 | 0 | 1 | 8 |
| Ertapenem | 30 | 0 | 0 | 30 |
| Erythromycin | 9 | 1 | 0 | 10 |
| Flucloxacillin | 8 | 0 | 0 | 8 |
| Fosfomycin | 13 | 0 | 0 | 13 |
| Fusidic acid | 8 | 0 | 0 | 8 |
| Gentamicin | 31 | 0 | 0 | 31 |
| Imipenem | 36 | 1 | 0 | 37 |
| Levofloxacin | 38 | 1 | 0 | 39 |
| Meropenem | 33 | 1 | 0 | 34 |
| Metronidazole | 1 | 0 | 0 | 1 |
| Nitrofurantoin | 13 | 0 | 0 | 13 |
| Oxacillin | 8 | 0 | 0 | 8 |
| Penicillin | 11 | 0 | 0 | 11 |
| Piperacillin-tazobactam | 35 | 2 | 3 | 40 |
| Rifampicin | 8 | 0 | 0 | 8 |
| Teicoplanin | 11 | 0 | 0 | 11 |
| Tetracycline | 9 | 0 | 1 | 10 |
| Ticarcillin-clavulanic acid | 2 | 0 | 0 | 2 |
| Tobramycin | 9 | 2 | 0 | 11 |
| Trimethoprim-sulfamethoxazole | 43 | 0 | 0 | 43 |
| Vancomycin | 14 | 0 | 0 | 14 |
| Total: | 734 | 15 | 10 | 759 |
